# Synthesis and Characterization of a Mg^2+^-Selective Fluorescent Probe

**DOI:** 10.3390/s140712560

**Published:** 2014-07-11

**Authors:** Chunwei Yu, Qiongyao Fu, Jun Zhang

**Affiliations:** 1 Laboratory of Environmental Monitoring, School of Tropical and Laboratory Medicine, Hainan Medical University, Haikou 571101, China; E-Mail: yucw_1979@sina.com; 2 Faculty of Laboratory Medicine, School of Tropical and Laboratory Medicine, Hainan Medical University, Haikou 571199, China

**Keywords:** Mg^2+^, fluorescent probe, Schiff base

## Abstract

A new Mg^2+^-selective fluorescent probe **P** was synthesized and characterized. With optimal conditions, the proposed probe **P** showed good selectivity to Mg^2+^ compared to other common metal ions, and worked in a wide linear range of 5.0 × 10^−7^–6.0 × 10^−6^ M with a detection limit of 1.7 × 10^−7^ M Mg^2+^ in ethanol-water solution (9:1, v/v, 20 mM HEPES, pH = 10.0).

## 1. Introduction

The magnesium ion (Mg^2+^) is the most abundant divalent cation in living cells, and participates in many important cellular processes, such as ion channel regulation, DNA and protein synthesis, membrane stabilization and cytoskeletal function [1,2]. Moreover, dietary deficiency of Mg^2+^ appears to play an etiological role in many diseases [3], thus, Mg^2+^ sensors are extensively required [4].

**Scheme 1.**
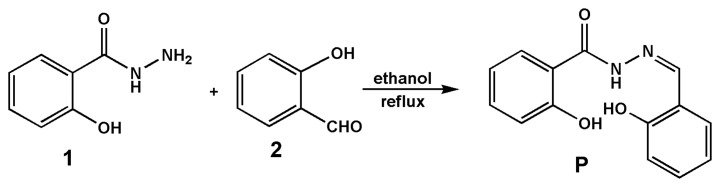
The synthesis route of probe **P**.

In recent years, different kinds of Mg^2+^-responsive fluorescent probes containing receptor groups based on moieties including crown ethers [2,4,5], calix[4]arenes [6], diketones [7,8], porphyrins [9] and imine-like aromatics [10–15] have been developed, and some of them are commercially available [16,17], but compared to the success of Ca^2+^-selective probes [18], the design of highly selective Mg^2+^ fluorescence probes is still an intriguing challenge. In this paper, we developed a compound by inserting an atomic spacer group between the carbon hydrazone coordinating sites to produce helicates with larger internal cavities, and thoroughly studied the affinity effect of coordination sites on Mg^2+^ selectivity (Scheme 1).

## 2. Experimental Section

### 2.1. Reagents and Instruments

All reagents and solvents are of analytical grade and used without further purification. The metal ions employed are NaCl, KCl, CaCl_2_·2H_2_O, MgCl_2_·6H_2_O, Zn(NO_3_)_2_·6H_2_O, PbCl_2_, CdCl_2_, CrCl_3_·6H_2_O, CoCl_2_·6H_2_O, NiCl_2_·6H_2_O, HgCl_2_, CuCl_2_·2H_2_O, FeCl_3_·6H_2_O and AgNO_3_, respectively.

Fluorescence emission spectra were conducted on a Hitachi 4600 spectrofluorimeter. UV-Vis spectra were obtained on a Hitachi U-2910 spectrophotometer. Nuclear magnetic resonance (NMR) spectra were measured with a Bruker AV 400 instrument and chemical shifts are given in ppm from tetramethylsilane (TMS). Mass (MS) spectra were recorded on a Thermo TSQ Quantum Access Agillent 1100 system.

### 2.2. Synthesis of Compound **P**

Compound **1** (1.0 mmol) and **2** (1.0 mmol) were stirred in ethanol (30 mL) at 80 °C for 6 h, and then cooled to room temperature. The precipitate so obtained was filtered and dried under vacuum and used directly. Yields: 87.6%. MS (ES+) *m/z*: 257.33 [M+H]^+^, 279.23 [M+Na]^+^. ^1^H-NMR (δ ppm, DMSO-*d_6_*): 12.05 (s, 1H), 11.79 (s, 1H), 11.22 (s, 1H), 8.69 (s, 1H), 7.91 (d, 1H, *J* = 7.40 Hz), 7.58 (d, 1H, *J* = 7.40 Hz), 7.46 (t, 1H, *J* = 7.38 Hz), 7.33 (t, 1H, *J* = 7.32 Hz), 7.00 (d, 1H, *J* = 8.12 Hz), 6.97 (d, 1H, *J* = 7.76 Hz), 6.95 (d, 1H, *J* = 4.52 Hz), 6.93 (d, 1H, *J* = 7.64 Hz). ^13^C-NMR (δ ppm, DMSO-*d_6_*): 165.43, 159.93, 158.43, 149.93, 134.88, 132.52, 130.40, 129.48, 120.30, 119.93, 119.53, 118.21, 117.36, 116.53.

### 2.3. General Spectroscopic Methods

Metal ions and probe **P** were dissolved in deionized water and DMSO to obtain 1.0 mM stock solutions, respectively. Before spectroscopic measurements, the solution was freshly prepared by diluting the high concentration stock solution to the corresponding desired concentration. For all measurements, excitation and emission slit widths were 10 nm, excitation wavelength was 385 nm.

## 3. Results and Discussion

### 3.1. The Effects of pH on **P** and **P** with Mg^2+^

**Figure 1.**
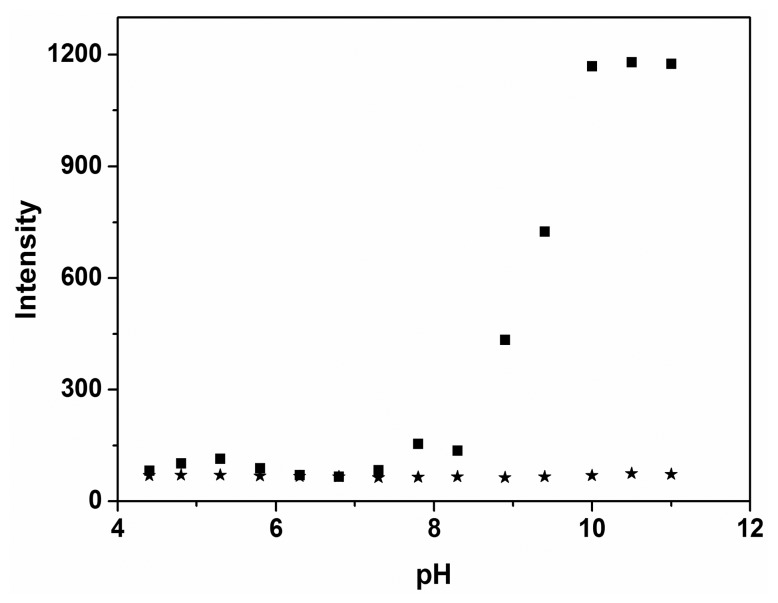
pH-dependence of **P** (10 μM) (★) and **P** (10 μM) plus Mg^2+^ (100 μM) (▪) in HEPES buffers as a function of different pH values in ethanol-water solution (9:1, v:v, 20 mM HEPES). The pH was modulated by adding 1.0 M HCl or 1.0 M NaOH in HEPES buffers.

A pH titration experiment was performed first to investigate a suitable pH range for the sensing of probe **P** to Mg^2+^. As shown in Figure 1, the emission intensities of the free probe **P** can be negligible in the range pH 4–11. After the addition of Mg^2+^ to the solution of probe **P** in the range of pH 4–11, the emission intensity at 460 nm rapidly increased to a maximum. For pH values smaller than 8.0, the emission intensity was significantly less than that for the high pH values, indicating poor stability of the Mg^2+^-**P** complexes at low pH. The pH-control emission measurements revealed that probe **P** showed the best respond to Mg^2+^ at pH 10.0. Therefore, further UV-vis and fluorescent studies were carried out in ethanol-water solution (9:1, v:v, 20 mM HEPES, pH 10.0).

### 3.2. UV-vis Spectral Response of **P**

**Figure 2.**
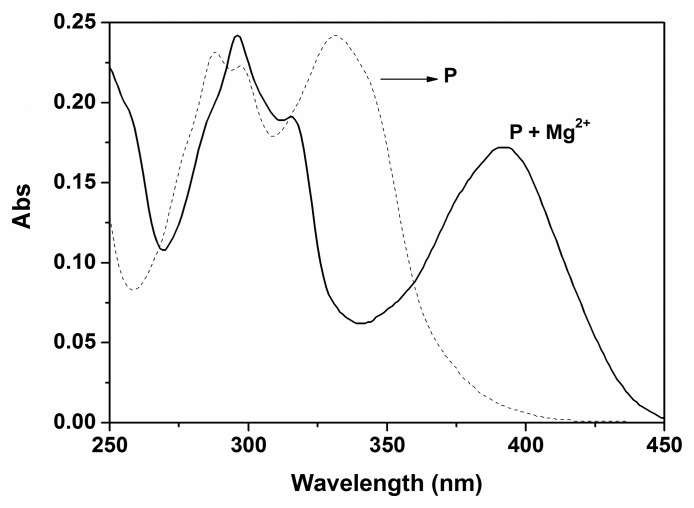
UV-vis spectra of **P** (10 μM) in ethanol-water solution (9:1, v:v, 20 mM HEPES, pH 10.0) upon addition of 100 μM Mg^2+^.

The addition of Mg^2+^ to the solution of **P** (10 μM) in ethanol-water solution (9:1, v:v, 20 mM HEPES, pH 10.0) caused an obvious spectra change in the UV region (Figure 2). This result in absorbance clearly suggested the binding of **P** with Mg^2+^. The new band at 395 nm suggested that Mg^2+^ binding with chemosensor **P** blocks conjugation between the double bonds, resulting in a longer absorption wavelength.

**Figure 3.**
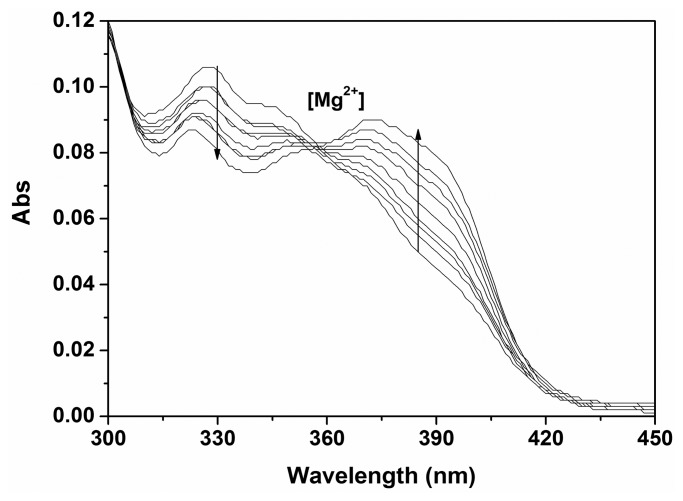
Absorbance spectra of **P** (10 μM) in ethanol-water solution (9:1, v:v, 20 mM HEPES, pH 10.0) in the presence of different amounts of Mg^2+^.

During Mg^2+^ titration with **P**, a significant decrease of absorption intensity at 327 nm and an increase of absorption band centered at 395 nm were observed with an isosbestic point at 356 nm, which indicated a clear ratiometric absoption change (Figure 3). These results indicated that **P** can function as an absorption ratiometric chemosensor for Mg^2+^ in aqueous media.

### 3.3. Fluorescence Spectral Response of **P**

**Figure 4.**
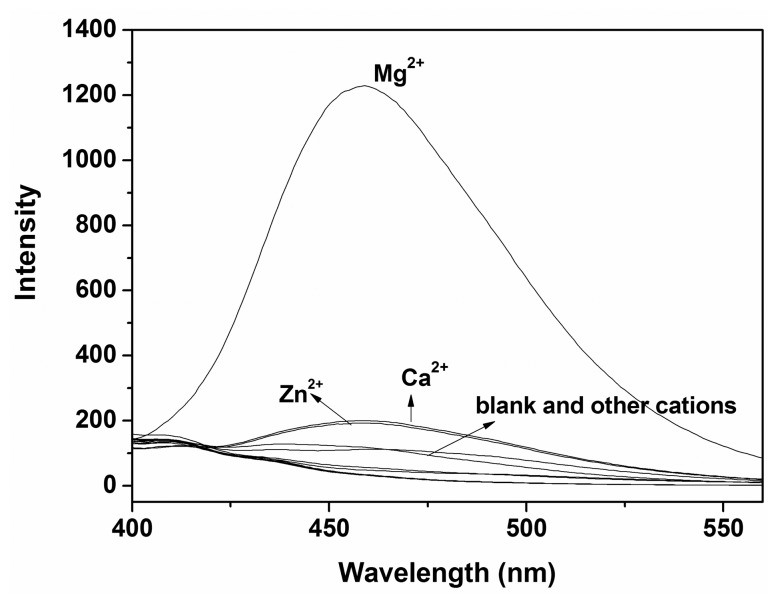
Fluorescence spectra of **P** (10 μM) with different metal ions (100 μM) in ethanol-water solution (9:1, v:v, 20 mM HEPES, pH 10.0).

To further evaluate the selectivity of probe **P**, the fluorescence spectra (Excitation wavelength was 385 nm) of **P** (10 μM) were investigated in ethanol-water solution (9:1, v:v, 20 mM HEPES, pH 10.0) with the addition of respective metal ions (100 μM) (Figure 4). Study showed that compared to other ions examined, only Mg^2+^ generated a significant “turn-on” fluorescence response of the monomeric peak at 460 nm with a fluorescence enhancement up to 56-fold. These results suggested that **P** had a higher selectivity toward Mg^2+^ than the other metal ions.

**Figure 5.**
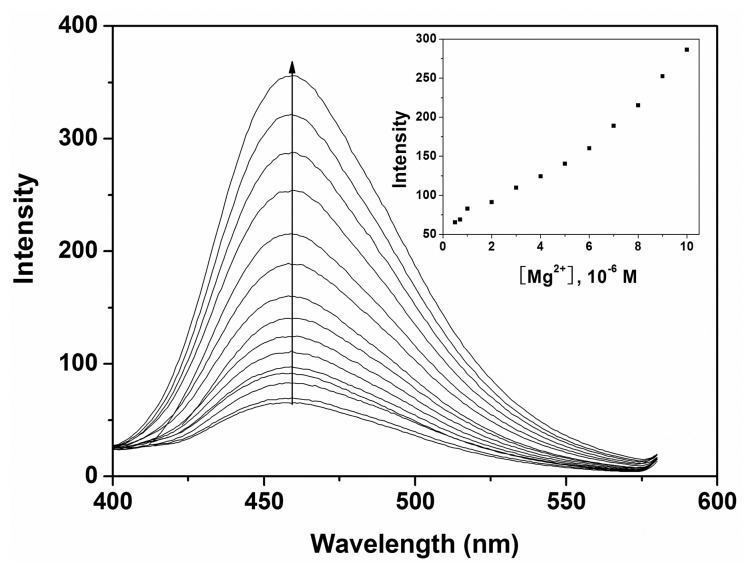
Fluorescence response of **P** (10 μM) with various concentrations of Mg^2+^ in ethanol-water solution (9:1, v:v, 20 mM HEPES, pH 10.0). Inset: the fluorescence of **P** (10 μM) as a function of Mg^2+^ concentrations (0.5–12 μM).

To further investigate the interaction of Mg^2+^ and **P**, a fluorescence titration experiment was carried out. The result showed that the fluorescence intensity of **P** was enhanced upon addition of various amounts of Mg^2+^ in ethanol-water solution (9:1, v:v, 20 mM HEPES, pH 10.0) as depicted in Figure 5. Under the present conditions, when **P** was employed at 10 μM level, the fluorescent intensity of **P** was proportional to the concentration of Mg^2+^ in the range of 1.3 × 10^−6^ to 1.0 × 10^−5^ M with a detection limit of 4.2 × 10^−7^ M Mg^2+^. This clearly demonstrated that probe **P** could sensitively detect environmentally relevant levels of Mg^2+^.

### 3.4. The Proposed Reaction Mechanism

**Figure 6.**
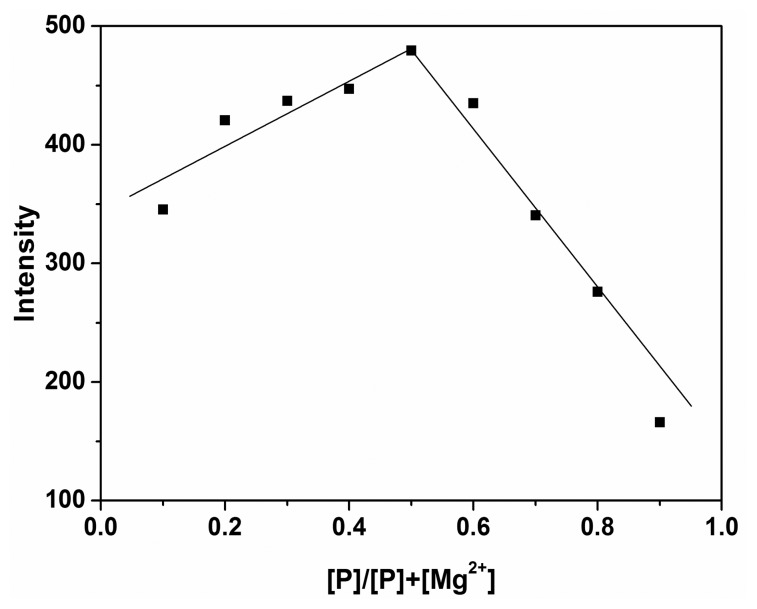
Job's plot for determining the stoichiometry of **P** and Mg^2+^. The total concentration of **P** and Mg^2+^ was kept 10 μM.

To study the reaction mechanism of **P** with Mg^2+^, the Job's plot experiment was carried out, and the result indicated that a **P**-Mg^2+^ complex was formed in 1:1 stoichiometry (Figure 6).

**Scheme 2.**
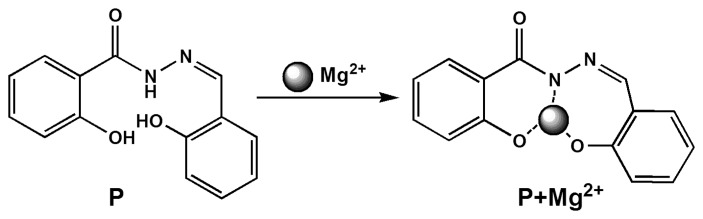
Proposed binding mode of **P** with Mg^2+^.

Thus, according to the obtained results, the reaction mechanism between **P** and Mg^2+^ was proposed. The probe **P** was most likely to chelate with Mg^2+^ as shown in Scheme 2, which blocked the photo induced electron transfer (PET) mechanism and greatly enhanced the fluorescence of the proposed probe.

## 4. Conclusions

In summary, a simple structure probe was synthesized facilely. This new fluorescent probe showed significant fluorescence enhancement in presence of Mg^2+^ in ethanol-water solution (9:1, v:v, 20 mM HEPES, pH 10.0). We believe that these observations should serve as the platform to develop new probes for other metal ions.
